# Prediction of In Vivo Laser-Induced Thermal Damage with Hyperspectral Imaging Using Deep Learning

**DOI:** 10.3390/s21206934

**Published:** 2021-10-19

**Authors:** Martina De Landro, Eric Felli, Toby Collins, Richard Nkusi, Andrea Baiocchini, Manuel Barberio, Annalisa Orrico, Margherita Pizzicannella, Alexandre Hostettler, Michele Diana, Paola Saccomandi

**Affiliations:** 1 Department of Mechanical Engineering, Politecnico di Milano, 20156 Milan, Italy; martina.delandro@polimi.it (M.D.L.); annalisa.orrico@polimi.it (A.O.); 2 Hepatology, Department of Biomedical Research, Inselspital, University of Bern, 3010 Bern, Switzerland; eric.felli@dbmr.unibe.ch; 3 Research Institute against Digestive Cancer (IRCAD), 67000 Strasbourg, France; toby.collins@ircad.fr (T.C.); manuel.barberio@ircad.fr (M.B.); Alexandre.Hostettler@ircad.fr (A.H.); michele.diana@ircad.fr (M.D.); 4 Research Institute against Digestive Cancer (IRCAD), Kigali, Rwanda; richard.nkusi@ircad.africa; 5 Department of Pathology San Camillo Forlanini Hospital, 00152 Rome, Italy; baiocchiniandrea@gmail.com; 6 Department of General Surgery, Ospedale Card. G. Panico, 73039 Tricase, Italy; margherita.pizzicannella@ihu-strasbourg.eu; 7 IHU-Strasbourg, Institute of Image-Guided Surgery, 67000 Strasbourg, France; 8 ICUBE Laboratory, Photonics Instrumentation for Health, University of Strasbourg, 67081 Strasbourg, France

**Keywords:** convolutional neural network, deep learning, hyperspectral imaging, infrared imaging, in vivo experiments, laser ablation, remote sensing, thermal damage, thermal damage prediction

## Abstract

Thermal ablation is an acceptable alternative treatment for primary liver cancer, of which laser ablation (LA) is one of the least invasive approaches, especially for tumors in high-risk locations. Precise control of the LA effect is required to safely destroy the tumor. Although temperature imaging techniques provide an indirect measurement of the thermal damage, a degree of uncertainty remains about the treatment effect. Optical techniques are currently emerging as tools to directly assess tissue thermal damage. Among them, hyperspectral imaging (HSI) has shown promising results in image-guided surgery and in the thermal ablation field. The highly informative data provided by HSI, associated with deep learning, enable the implementation of non-invasive prediction models to be used intraoperatively. Here we show a novel paradigm “peak temperature prediction model” (PTPM), convolutional neural network (CNN)-based, trained with HSI and infrared imaging to predict LA-induced damage in the liver. The PTPM demonstrated an optimal agreement with tissue damage classification providing a consistent threshold (50.6 ± 1.5 °C) for the damage margins with high accuracy (~0.90). The high correlation with the histology score (r = 0.9085) and the comparison with the measured peak temperature confirmed that PTPM preserves temperature information accordingly with the histopathological assessment.

## 1. Introduction

Thermal ablation is a minimally invasive alternative treatment for primary liver cancer with lower morbidity, reduced cost, and shorter hospitalization times compared to the surgical approach [1,2]. This technique induces local tissue necrosis by applying extreme temperatures inside the tumor nodule for a short time. Radiofrequency ablation, microwave ablation, and laser ablation (LA) are the most common thermal procedures available. Among them, LA has demonstrated its value for treating liver tumors since 1966 [3]. Due to the small dimension of the laser applicator (<1 mm), LA is one of the least invasive tools in high-risk or difficult to reach tumors locations. Additionally, the short procedure time makes LA a very promising minimally invasive technique [4,5]. Furthermore, in 2015, Di Costanzo et al. found that LA showed the efficacy of tumor ablation, time to local progression, and overall survival not inferior to radiofrequency ablation [6]. Although the procedure holds the mentioned benefits and is currently in the spotlight, also thanks to the combination with promising tools, such as the nanoparticles-mediated photothermal therapy [7] and the immunotherapy [8], some shortcomings are still present. Indeed, usually clinicians rely on predetermined dosimetry without any thermal damage quantitative feedback during the LA procedure. This approach lacks standardization and consistent monitoring, which may cause unsatisfying therapeutic outcomes [9].

Aiming to address this limitation, several thermometry systems for tumor thermotherapies have been proposed in the last decades, such as temperature sensors and magnetic resonance temperature imaging (MRTI) [10,11]. Concerning the temperature sensors, thermocouples are widely used because of cost-effectiveness and small size. Nevertheless, they are invasive devices providing only a single-point measurement. Additionally, their metallic conductors can lead to a significant overestimation of the actual temperature [12]. Further innovative monitoring techniques using fiber optic sensors are under study [13,14]. They allow the measurement of temperature in different points of the tissue with excellent time resolution, but their approach still requires contact with the tissue and provides sparse temperature information. Thus, when available, the sensor-based control of the treatment relies only on the temperature feedback in a few tissue regions, without thermal damage monitoring. In MRTI scenario, the thermal damage is usually obtained by simplified models relying on the measured temperature and exposure time, like the Arrhenius integral [15]. The map of damage is reconstructed from the temperature images produced with MRTI. The software displays temperature and damage in multiple image planes, enabling the visual control of the ablative margins during the treatment [16]. However, the correlation between the thermal damage calculated with the model and the actual damage induced in the organ is still under investigation [17]. Hence, a degree of uncertainty remains on the ablated safety margins and treatment effect due to some limitations of both the Arrhenius model and MRTI technique. The simplifications of the Arrhenius model, such as the hypothesis of the first-order kinetics for protein denaturation, may potentially cause inaccurate thermal outcome estimations [18]. Additionally, the effectiveness of the Arrhenius model in determining thermal damage hinges on accurate temperature measurements. Artifacts in the MRTI images may strongly affect temperature estimation, thus causing errors in the thermal damage prediction [19,20]. Furthermore, MRTI is costly and holds important limitations related to the necessity of working with MR-compatible devices. Recently, optical techniques have been investigated to directly assess the induced thermal damage. These approaches have shown promise in differentiating normal and thermal damage tissue by measuring tissue optical properties [21]. Indeed, both scattering and absorption tissue properties are affected by laser-induced thermotherapy [22]. In 2020, Nagarajan et al. proved that the relative changes in scattering and absorption can be used to classify thermal damage scores with an overall accuracy of 72.5% at temperatures up to 75 °C [23]. Diffuse reflectance spectroscopy has been shown to be a promising approach to monitor the tissue thermal variation intraoperatively at the tip of a fiber-optic needle (i.e., spectroscopy needle) using the light reflected from the therapy target [24,25]. Additionally, it allows for highly accurate discrimination between ablated and non-ablated animal liver tissue during radiofrequency ablation, but only in a few locations. Therefore, optical technology is a promising tool for achieving safe ablation margins [26,27]. Among the optical approaches, hyperspectral imaging (HSI) recently gained importance in image-guided surgery [28,29] and in the ablative therapy field [30,31,32]. HSI provides a discrete three-dimensional image with two spatial dimensions (x,y) coupled with a third dimension (z), which gives the relative reflectance of each pixel of the image within a narrow spectral band, forming a hypercube. The highly informative data provided by HSI, coupled with machine learning and particularly deep learning, make the implementation of non-invasive prediction models possible intraoperatively [33,34]. In 2018, Guan et al. demonstrated the ability of autofluorescence-based HSI to detect lesions using an unsupervised learning method to optimize the radiofrequency treatment [35]. Contrary to MRTI, HSI provides a cost-effective instrument and does not require compatible devices. Furthermore, the HS technology guarantees robust data of the texture of the biological tissue, and the structural alteration induced by LA treatment.

In light of the need for intraoperative LA monitoring and considering the current limitations of the existing alternatives, here we propose an HSI-based approach using a novel paradigm “peak temperature prediction model” (PTPM) for the monitoring of thermal damage during LA. PTPM is implemented with a convolutional neural network (CNN) trained with hyperspectral (HS) and infrared (IR) imaging data. Using the tissue spectral signature, PTPM is aimed to automatically predict peak temperature maps holding thermal damage information during LA.

## 2. Materials and Methods

### 2.1. Study Design

**Figure 1:**
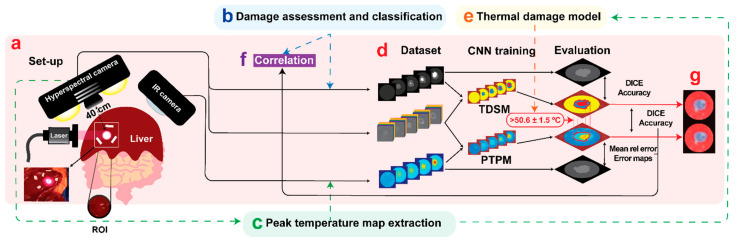
(**a**) The experimental setup. The laser ablation procedure was performed on the in vivo pig liver surface. The ablation progress was recorded by HS and IR cameras. A circular ROI centered on the ablation spot was defined in the images and used in the CNN implementation. The data collected during the experiment were used to obtain the final dataset for training the TDSM and PTPM. (**b**) TDSM dataset creation. Starting from the damage assessment of the histology, RGB images collected from the HS camera were manually annotated. (**c**) PTPM dataset creation. Extraction of peak temperature maps from the IR camera video. (**d**) The TDSM was trained with supervised learning using hypercubes and damage zone annotations obtained from the RGB images. The PTPM was trained with supervised learning using the hypercubes and peak temperature maps extracted from IR video. The two models were trained and tested using pixels in the ROIs. (**e**) Peak temperature maps were used as a model for thermal damage outcome. (**f**) The PTPM was validated by applying a linear correlation with histology. (**g**) As a final step, damage predictions of the two models were compared and the PTPM was validated also using TDSM results. The area of damage in the predicted peak temperature maps was obtained using the threshold of 50.6 ± 1.5 °C.

The overall design of our study is reported in Figure 1. An in vivo liver LA was performed with a contactless approach on the pig liver surface and the ablation progress was recorded by HS and IR cameras (Figure 1a). After identifying three classes of damage in the histology assessment, “no damage”, “ring”, and “thermo”, red, green, and blue (RGB) images were annotated (Figure 1b), and peak temperature maps were extracted from the data collected with IR camera (Figure 1c). The HS ability to predict the thermal damage was investigated by developing two CNN models (Figure 1d): (i) tissue damage segmentation model (TDSM) which automatically categorizes (or classifies) pixels in a selected region of interest (ROI) (Figure 1a) into the three classes, and (ii) peak temperature prediction model (PTPM) that, for each pixel in the ROI, predicts the maximal (peak) temperature that has been reached during the overall ablation procedure at that pixel. Whereas the first model provides a coarse-grained prediction of the induced damage, the second one produces a finer-grained assessment of the thermal damage during the procedure. Indeed, the PTPM thermal damage model was based on the measured peak temperature maps (Figure 1e). The justification of this choice is reported in the Appendix A. HS potential in distinguishing the levels of thermal damage was finally validated by applying a linear correlation between histology scores and the peak temperature measured and predicted for the three classes (Figure 1f). Finally, the results of the two models were matched to verify the consistency of the predicted margins of LA-induced damage (Figure 1g).

### 2.2. Experimental Strategy

**Figure 2:**
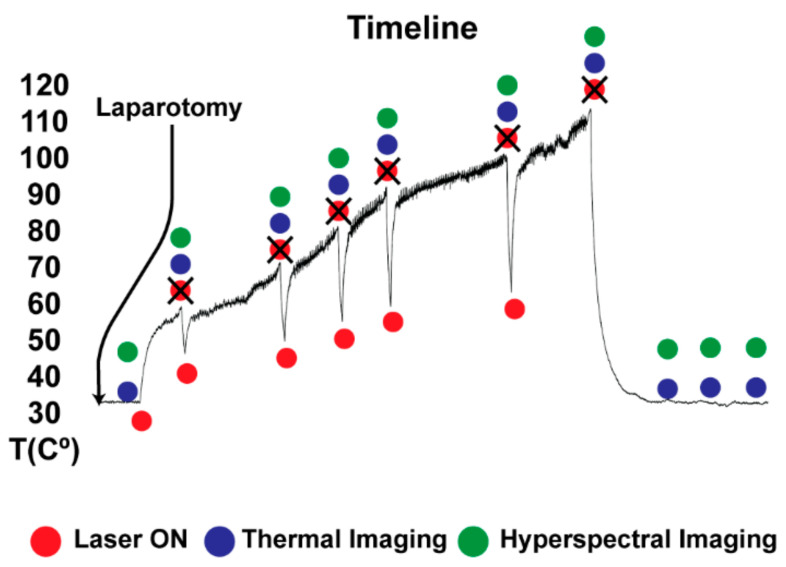
After laparotomy, liver tissue was irradiated until specific temperature thresholds occurred. The temperature was monitored continuously using the IR video. Once the set temperature threshold was reached, the laser system was switched off and the HS data (hypercube and RGB image) were acquired.

The study is part of the LASER OPTIMAL ERC project (GA n. 759159), approved by the local Ethical Committee on Animal Experimentation (ICOMETH No. 38.2015.01.069), as well as by the French Ministry of Superior Education and Research (MESR) (Protocol n° APAFiS##19543-2019030112087889, approved on the 14 March 2019). All animals used in the experiment were managed according to: (i) ARRIVE guidelines, (ii) French laws for animal use and care, and (iii) the directives of the European Community Council (2010/63/EU). Two adult swine (Sus scrofa ssp. domesticus, mean weight: 33.5 ± 4.9 kg) were housed for 48 h in an enriched environment, with constant humidity and temperature, respecting circadian cycles of light-darkness. All the animals were fasted 24 h before surgery, with ad libitum access to water and sedated with zolazepam and tiletamine 10 mg/kg IM 30 min before the procedure. The anesthesia was performed with Propofol 3 mg/kg (18-gauge IV catheter in ear vein) and maintained with rocuronium 0.8 mg/kg along with isoflurane 2%. Vital parameters were monitored through a standard respiratory machine (Primus, Dräger, Drägerwerk AG & Co. KGaA, Lübeck, Germany). Midline laparotomy was performed as well as the mobilization of the liver into the central axis of the abdomen. Six distinguished areas on the liver surface underwent contactless LA (two in the first pig and the remaining four in the second pig). LA was performed by diode laser (LuOcean Mini 4, Lumics, Berlin, Germany) delivering the radiation to a 400 μm fiber applicator. A collimator was placed on the applicator tip to convey an 808 nm laser light on the tissue surface. The collimated beam diameter was around 1.5 cm, and the laser current was tuned from 3000 to 3300 mA. Liver tissue was irradiated until specific temperature thresholds occurred (Figure 2). The temperature was monitored using the IR thermographic camera (FLIR T540, 464 × 368 pixels spatial resolution, 2 °C accuracy) capturing images of the scene at 10 fps. Temperature maps of the liver surface undergoing ablation were used firstly to monitor the procedure. An ROI was placed in the ablated area to monitor in real-time the maximum temperatures reached in the target (i.e., 60, 70, 80, 90, 100, 110 °C). These temperature thresholds were used as indicators of produced thermal effect. Once the set temperature threshold was reached, the laser system was switched off and the HS data were acquired. A hypercube and an RGB image were collected from the HS camera for each acquisition step. In the postprocessing phase, 10 IR images were extracted from the IR video of the treatment and used in the following steps of the work. A total of 10 hypercubes, 10 RGB, and 10 IR images were used in the analysis of each ablation. They were associated with the superficial tissue temperature (36 °C), the six threshold temperatures, and 1, 3, and 5 min after the procedure. To minimize the source of error for the IR temperature, humidity, and air circulation of the imaging environment were controlled during the whole experiment. The measurement conditions were as follows: air humidity 50–55%, air and ambient temperature 25 °C, reflective temperature 25 °C. The emissivity parameter was set at 0.95 [36]. At the end of the procedure, the animals were euthanized with a lethal dose of pentobarbital (40 mg/kg) (Exagon, Axience, Pantin, France), under a 5% isoflurane anesthesia.

### 2.3. Hyperspectral Imaging

The HS camera system (TIVITA, Diaspective Vision GmbH, Am Salzhaff, Germany) acquired hypercubes (640 × 480 × 100 each) and RGB images for ten acquisition steps. The hypercube was acquired in around 6 s and synchronized with the absence of breathing motion accordingly with a protocol implemented for the animal anesthesia. Markers of polyurethane material were placed around the target area and used as references for overlaying the HS and IR images. The target areas were chosen under manufacturer guidelines of the HSI camera use. The TIVITA camera was perpendicularly adjusted with a 40 cm distance minimizing external light irradiations on the measuring area [37]. Additionally, all the light sources in the surgery room, except for the camera’s lamps, were switched off during the HS acquisition. The HS camera is equipped with a push-broom imaging spectrometer with a slit-shaped aperture, internal stepper motor moving the slit of the spectrograph, high quality IR enhanced CMOS, and data processing equipment. The acquisition of a single hypercube is performed with a camera-specific module of the Perception Studio software (Perception Park GmbH, Graz, Austria). The spectral range of this camera is 500–995 nm. The light source is a 20 W OSRAM Halospot 70 Halogen lamp allowing for intense, broadband, temperature-stable, homogeneous, and fast pulses radiation. The calibration of the wavelength is performed during camera production. Furthermore, dark current effects are corrected after the recording of the data cube by the developed software component. The camera collects and processes the information from the electromagnetic spectrum measuring the reflectance spectra generated by the object of study. To convert image data from radiance to relative reflectance, a white reference object characterized by a high diffuse reflectance is used to create a reference cube before the measurements started.

### 2.4. Convolutional Neural Networks

This work used two CNNs to predict thermal damage from the HSI hypercubes acquired during the ablation procedure. The architectures of the CNNs were very similar and based on Hamida et al. [38], which has shown excellent performance for HSI classification with remote sensing data [39]. In this section, we describe the function of each CNN and how they were trained. The implementation details of the CNN architectures are also provided at the end.

#### 2.4.1. Tissue Damage Segmentation Model (TDSM)

The TDSM was implemented with a classification CNN trained using supervised learning. Specifically, the TDSM was trained to take as input the HS information and to output the classification of the pixel into “no damage”, “ring”, and “thermo”.

**Figure 3:**
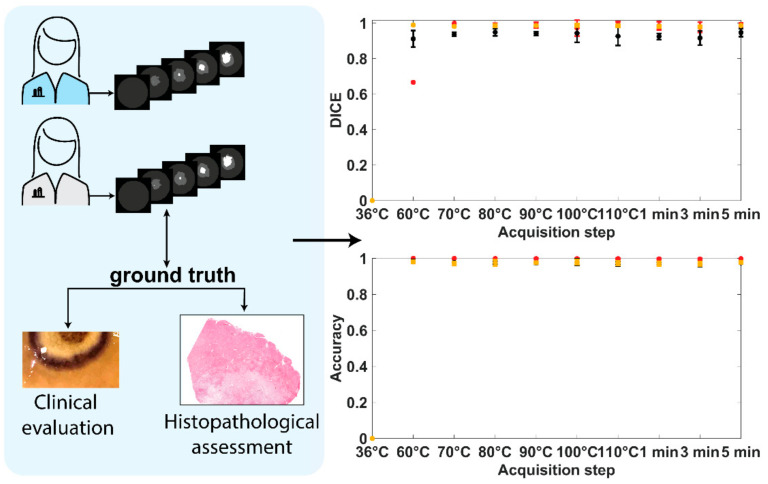
TDSM dataset creation. The manual segmentation was performed by visual inspection based on the color profile. Two annotators were used, and inter-annotator agreement was assessed with DICE and accuracy scores.

Dataset creation: Training the CNN required creating a suitable dataset, then using it to train and test the model. The first HS image was taken as a reference image for the superficial tissue temperature. A circular ROI was defined in the reference image centered on the ablation spot (Figure 1a), which was made sufficiently wide to encompass all thermally damaged tissue during the procedure. The mean ROI diameter was 58 px. Using the optical markers, the ROI was spatially aligned with all HS images. This was achieved using an affine image alignment computed using Matlab’s fitgeotrans and imwarp functions. After the alignment, the location of each ROI in each HSI was known. From each HSI, the corresponding RGB image was then manually annotated by two trained surgeons using GNU Image Manipulation Program into three classes in the images: “no damage”, “ring”, and “thermo”. The manual segmentation was performed by visual inspection based on the color profile. To assess the objectivity of the annotations, two annotators were involved, and the inter-annotator agreement was assessed. Results are reported in Figure 3. DICE and accuracy were set as metrics to assess the inter-annotator agreement. They are standard metrics to validate the image segmentation algorithm and enable complementary evaluation. In particular, the accuracy represents the percentage of correctly predicted pixels in the images, and the DICE coefficient is a measure of segmentation overlap. Results show values higher than 0.97 and 0.91 for DICE and accuracy, respectively, indicating very high agreement, and thus justifying the use of the RGB annotated images as reference for the damage classes. For the DICE at 60 °C the lowest value of 0.66 is due to the “thermo” small area to depict, thus a few pixel errors hardly affect the final parameter.

Training: The first annotator’s segmentations were used to train the TDSM using supervised learning with 6-fold cross-validation. It was implemented in Pytorch and trained with backpropagation using Stochastic Gradient Descent optimization, the inverse frequency cross-entropy (MAE) loss function, a learning rate of 0.001, and a batch size of 1. Training was terminated after 5 epochs (a total of 287 training iterations). Training was conducted on a standard desktop workstation PC (PRIME Z390-A) using an Nvidia RTX 2080 graphics card, requiring approximately 2880 min of training time.

Evaluation: Multi-class DICE and accuracy were used as metrics to evaluate the performances of the TDSM. The two metrics were calculated between the pixels in the ROI of ground truth and predicted images at each acquisition step and for each ablation test. Pixels belonging to specular reflection areas were excluded from the ROI in the evaluation of the performance because they convey no HS information. Mean and the standard deviation were measured among the tests to give a single metric result at each acquisition step. The standard deviation is showed for the acquisition step with n ≥ 3.

#### 2.4.2. Peak Temperature Prediction Model (PTPM)

The PTPM was implemented with a regression CNN trained using supervised learning. Specifically, the CNN was trained to take as input the HS information and to output the predicted peak temperature at the given pixel (°C). 

**Figure 4:**
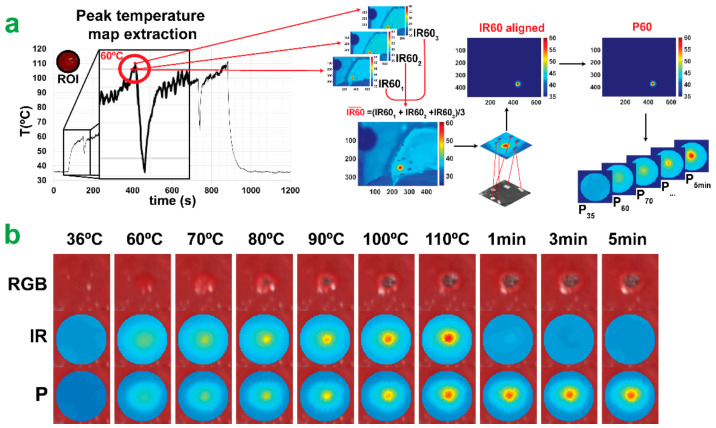
Peak temperature map extraction. (**a**) PTPM dataset creation. Extraction of peak temperature maps (P_*i*_) from IR camera video. (**b**) The normal RGB is reported with IR_*i*,*aligned*_ and P_*i*_ images for each acquisition step.

Dataset creation: Training the CNN required creating a suitable dataset, then using it to train and test the model. The dataset was created from the HS and IR camera data of each experiment. IR images were extracted from the IR video, recording the overall procedure, to match the hypercubes acquisition step. An example of the process is reported in Figure 4a for the IR image at 60 °C. The first step was the extraction of the thermal images from the IR camera video. A single characteristic temperature map was extracted for each acquisition step (IR_*i*_) averaging ~5 IR images. The 10 IR and 10 HS images were then spatially aligned to compensate for two factors: the spatial separation of the cameras and potential physiological movements between time points due to respiration. A spatial alignment was needed to associate each pixel in the HS image with its corresponding temperature value at all time points. The spatial and temporal alignment was achieved using 5–6 optical markers that were fixated in each ablation zone, which were visible in both the HS and IR images. The centers of each optical marker were carefully marked in each of the 10 images for each ablation zone. The same ROIs in the HS images used in the TDSM were spatially aligned with IR images using Matlab’s fitgeotrans and imwarp functions. The aligned IR images were labeled as IR_*i*,*aligned*_. After the alignment, the peak temperature maps were constructed. The peak temperature map P_*i*_ at acquisition step *i* was computed by evaluating the maximum temperature among all aligned thermal images between times 1 to *i* (inclusive) within the ROI. Final P_*i*_ images are reported in Figure 4b.

Training: The input of the PTPM was the spectral measurements at and around a given pixel in the ROI, and its output was a real-valued number in °C (peak temperature for that pixel). The Pi was used to train the PTPM with supervised learning. We emphasize that although the dataset consisted of 60 HSIs, each pixel in the ROI was used to train the CNN, generating 148,877 possible training samples, which was sufficient for deep learning. An important aspect was how to train the CNN and to evaluate its ability to predict values on new data (i.e., HSIs that were not used for training). To achieve this purpose, cross-validation was applied. Specifically, the CNN was trained on data from 5 of the 6 ablation procedures, then its predictive performance was tested using data from the held-out ablation procedure. To maximize the use of data, this process was repeated 6 times, so that in each repetition, data from one ablation procedure were held-out testing and the remaining data were used for training (6-fold cross-validation, also used to train and test the TDSM). The CNN was implemented in Pytorch and trained with backpropagation using Stochastic Gradient Descent optimization, the Mean Relative Error loss function, a learning rate of 0.001 and a batch size of 1. Training was terminated after 5 epochs (a total of 115 training iterations). Training was conducted on a standard desktop workstation PC (PRIME Z390-A) using Nvidia RTX 2080 graphics card, requiring approximately 2880 min of training time.

Evaluation: To evaluate the performances of the PTPM a relative error percentage (Rel_*err*_) was measured as follows:

(1) $${\mathrm{Rel}}_{err}=\left.\left|\frac{P_{i,predicted}-P_{i,actual}}{P_{i,actual}}\right.\right|\cdot 100$$

The predicted temperature maps (P_*i*,*predicted*_) were compared with ground truth maps (P_*i*,*actual*_) and a pixel-pixel difference between the peak temperature values was computed in the analyzed ROI. 2D maps of Rel_*err*_ were obtained for each acquisition step and they are shown in the Results Section for the three classes: “no damage”, “ring”, and “thermo”. To select pixels in each class, ground truth classes of the annotated RGB images were used. Additionally, for each acquisition time and ablation test, a single mean relative error (MRE) % was measured by averaging the pixels in the three zones of interest. Finally, mean and standard deviation were computed among the tests to give one characteristic MRE % for each acquisition step. The standard deviation is showed for the acquisition step with n ≥ 3.

#### 2.4.3. CNN Architectures

The input to the CNNs was a hyperspectral l × w × w sub-volume centered on each pixel in the ROI with a spatial width and height of w pixels and l spectral bands. We used all spectral bands from the camera with l = 100, and w = 5 pixels. A relatively small spatial window of 5 pixels was used for two reasons. Firstly, it meant that the CNNs had a relatively small number of trainable parameters (see below), allowing them to be trained from scratch and end-to-end with limited training data. Secondly, it ensured the CNN to focus more on learning relevant spectral features without relying on significant spatial context information. In real-world applications, this context information may not be available or unreliable because of occlusions and limited camera field-of-view. Consequently, the performance of this CNN indicated the capability to predict LA-induced damage based primarily on spectral information. The architectures of the TDSM and PTPM CNNs are provided in Table 1. The architectures were identical except for the last fully connected (FC) layer. The TDSM had three output neurons, corresponding to the three damage classes. The PTPM had 1 output neuron, corresponding to the predicted peak temperature value. The kernel shape and strides associated with the convolutional layers (Conv1, Conv2, and Conv3) and pooling layers (Pool1, Pool2, and Pool3) are provided as set of three values (spectral dimension z, spatial dimension x, and spatial dimension y). The CNNs transformed the input sub-volume by a sequence of convolutional layers (Conv1, Conv2, Conv3, and Conv4) that extracted 3D feature maps. The convolutional layers had gradually larger receptive fields in the spectral domain, using intermediate pooling layers (Pool1, Pool2, and Pool3) that were implemented with 1D convolutional filters with a stride of 2 in the spectral domain. ReLU activation layers were used before each pooling layer to supply non-linearity. The total number of trainable parameters for the TDSM and PTPM were 31,988 and 31,076 respectively. The TDSM had 912 more trainable parameters than the PTPM because it used three fully connected output neurons compared to the PTPM which had one output neuron.

### 2.5. Histology

Liver biopsies were sampled at the end of the procedure in the spot of the laser treatment including the surrounding healthy tissue. Biopsies were cut for frontal and intraparenchymal staining. Formalin-fixed paraffin-embedded sections of 5 μm were stained using Harris Hematoxylin formula (Leica Biosystems). Two histopathological scores were assigned by a pathologist in a blinded fashion for the analysis of the Glisson’s area and the parenchyma respectively. The first score was based on cell necrosis, congestion, stromal oedema (scale: (0) none, (1) mild, (2) moderate, and (3) severe) and sinusoidal dilatation (scale: (0) healthy, (1) altered). The second score was based on Glisson’s detachment, inflammatory elements infiltrations, collagen damage (scale: (0) none, (1) mild, (2) moderate, and (3) severe) and mesothelium damage (scale: (0) healthy, (1) altered or absent). The score analysis was applied within the diameter of the LA-induced damage and outside in the healthy parenchyma.

Statistics: Statistical analysis was performed with GraphPad 8.3 (Prism, GraphPad Software, San Diego, CA, USA). Pearson’s rho was applied to correlate predicted and measured temperature with the histology score calculated in the Glisson’s capsule. Repeated One-way ANOVA with Dunnett’s and Tukey multiple comparisons tests were performed accordingly with the assumption of the data distribution. A two-tailed analysis with *p* value < 0.05 was considered statistically significant.

## 3. Results

### 3.1. Histological Damage Classification

**Figure 5:**
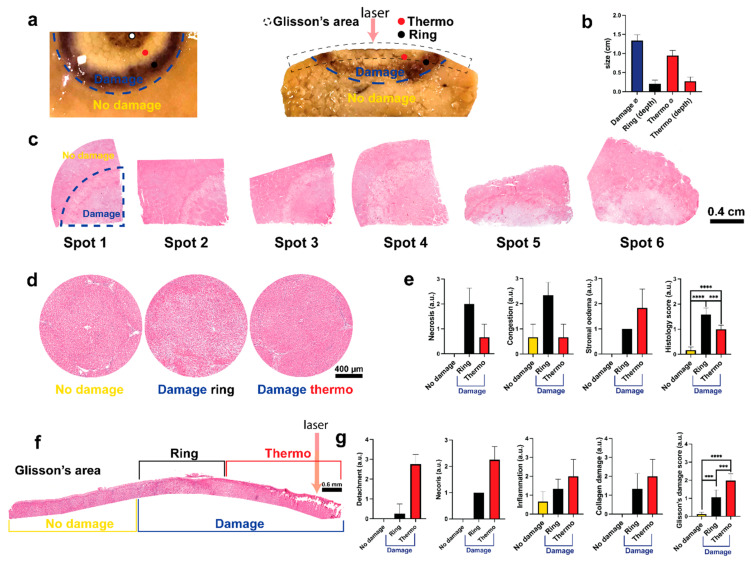
(**a**) Figure target of the biopsy, frontal and intraparenchymal view. Two classes, “damage” and “no damage”, are visible. Within the “damage” class it is possible to observe two classes: one homogeneous called “ring” and one heterogeneous called “thermo” composed by a white area visible in both views and one by a carbonization which characterizes only the Glisson’s area. (**b**) Descriptive analysis of the damage size. (**c**) Frontal view of all the spots. Spots 1 to 4 pig 1, spots 5 and 6 for pig 2. The margin of the damaged area is visible accordingly with the figure target. (**d**) Macro of the 3 main classes reconstructed with 5 to 10 images 10×. Compared with the “no damage” area, “ring” and “thermo” present a reperfusion and ischemic damage given by the heating transferred by the laser from the Glisson’s to the parenchyma in a radial fashion. (**e**) Histology score of the parenchymal view. Necrosis, congestion, and stromal oedema are displayed separately and their averages with the sinusoidal dilatation are shown on the right. The three areas are statistically different. (**f**) Glisson’s area map reconstructed with 9 images 10×. “Thermo” class presents a visible detachment with coagulative necrosis and superficial carbonization of the collagen and parenchyma. (**g**) Score of the Glisson’s area. Detachment, necrosis, inflammation, and collagen damage are shown separately. Glisson’s damage score represents the average with mesothelium damage. The three areas are statistically different. One-way ANOVA was used. Data are presented as mean ± s.d. and compared to the control, two tailed *p*-value *p* ≤ 0.05 was considered statistically significant, *** *p* ≤ 0.001, **** *p* ≤ 0.0001. N = 6 (ROIs in 2 pigs). Pictures sampled with microscope Zeiss AXIO scope A1.

The figure target of the biopsy presents a visible and well-delineated area of damage (Figure 5a). We defined “damage” and “no damage” classes accordingly. Overall, the damaged space is heterogeneous with a brown to black area due to the carbonization surrounded by a peripheral area with normal parenchymal lobule. We classified the first area as “thermo”. Following, the second area is more homogeneous and surrounds the entire “thermo” class delineating the margin of the whole “damage” class. We classified this area as “ring”. Descriptive analysis of the damage shows a consistent standard deviation between the spots with a similar depth of “thermo” and “ring” area (Figure 5b). All the spots present the same radial pattern of the burdens of the damage (Figure 5c).

The magnification of the three classes is shown in Figure 5d. The “no damage” class presents hepatic lobules with healthy radial sinusoids and central vein. Sinusoids gradually increase the diameter while they converge to the central vein. Kupffer cells and endothelial cells are visible with a large vein branch in the portal tract characterized by a normal arterial branch. Nuclei of endothelial cells appear protruding with a normal bile duct that shows a single layer of cholangiocytes and a preserved stroma. 

In the “ring” class it is possible to observe the dilatation of the sinusoids and the central vein. Hepatocytes present intercellular detachment with the loss of the normal hepatic trabeculae. Hepatocyte’s cytoplasm presents increased eosinophilia with a centric localization of the nuclei. Necrosis of single cells is present. The stroma appears oedematose in the portal tract and in the septa. This class presents common aspects with both congestion and reperfusion damage in the internal and external sides of the “ring” respectively.

The “thermo” class shows sinusoidal collapse with slight congestion. Additionally, the increasing of the hepatocytes volume and the homogenization with the pale staining of the cytoplasm suggest an ischemic-like damage. Infiltration of inflammatory mononucleate cells is present at the intraparenchymal level with moderate stromal oedema.

The histological score showed higher necrosis and congestion in the “ring” while stromal oedema showed a gradual increase (radial decrease). The difference between the three classes is statistically significant: “no damage” 0.1667 ± 0.1291, “ring” 0.1583 ± 0.2582 (*p* < 0.0001 vs. “no damage”), “thermo” 1.000 ± 0.1581 (*p* < 0.001 vs. “no damage”), (*p* = 0.0002 “ring” vs. “thermo”) (Figure 5e).

The action of the laser was observed superficially. Glisson’s area is shown in Figure 5f. In the “no damage” class, Glisson’s capsule presents a normal thickness involved by the mesothelium with a compact connective tissue and regular micro and macrovascular structure. A slight sub-capsular oedema is present probably due to the multiple spot damage over the same liver. 

The capsule is thicker in the “ring” class due to the oedema which separates collagen fibers producing a “basket-wave” like aspect. The mesothelium is dissolved, homogenized fibers are present with an initial sub-glissonian detachment. Moderate presence of inflammatory elements mono and polymorphonucleated are accumulated in the inter and sub-capsular space. Glisson’s capsule is thickened and interrupted in several points with a clear detachment. The surface is irregular with a homogenized connective tissue fused with plasma without the mesothelium. Accumulation of inflammatory elements is visible in the sub-capsular space. Sub-glissonian hepatocytes are detached from the trabeculae with a marked acidophilic degeneration of the cytoplasm. The septa that start from the Glisson’s capsule are oedematose which small arterial branches contracted and thickened leiomuscular wall. The deep parenchyma presents a transition zone in which morphological characteristics of the hepatocytes are similar to the ischemic damage, accordingly with the intraparenchymal observation made above. 

The histological score of all the elements presents a gradual increase from the “no damage” to the “thermo” class (Figure 5g). The increase in “ring” and “thermo” class is statistically significant when compared to “no damage” score of 0.133 ± 0.103 (*p* = 0.0006 vs. “ring”, *p* < 0.0001 vs. “thermo”), “ring” 1.050 ± 0.4195 (*p* = 0.0006 vs. “thermo”), and “thermo” 1.975 ± 0.3738.

### 3.2. Damage Prediction and Margins Detection

**Figure 6:**
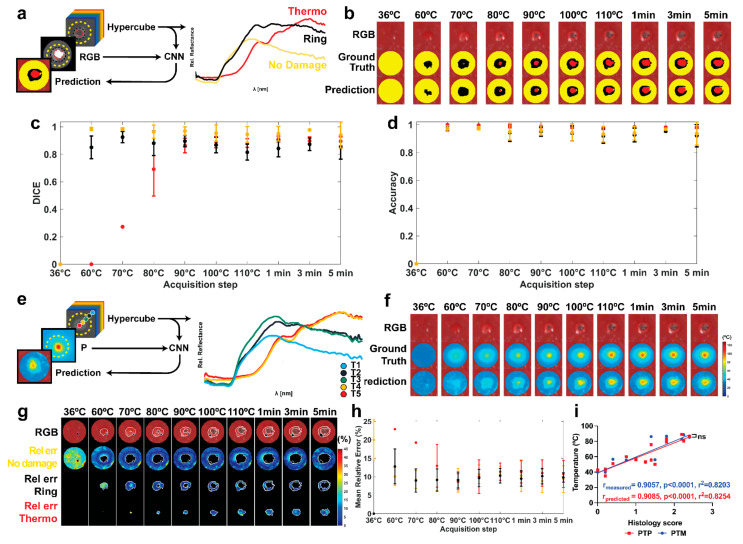
(**a**) Dataset and output of the TDSM, and relative reflectance for each class. (**b**) The normal RGBs are shown with ground truth and predicted pixel classes within the ROI of the first test. (**c**) Mean DICE scores for the three classes with standard deviation for all the acquisition steps. (**d**) Mean accuracy was also measured with standard deviation. (**e**) Dataset and output of the PTPM, and relative reflectance for several pixels associated with specific temperature value (T_1_ … T_5_) provided by the IR camera. (**f**) The normal RGBs are shown with ground truth and predicted peak temperature maps within the ROI of the first test. Predicted and ground truth temperature values, in the range 0–125 °C, are overlayed to the correspondent RGB images. (**g**) Maps of the Rel_err_ (%) for the three classes. Damage class borders are highlighted in white on the RGB images. The black spot shows the pixels selected as specularity in the images. (**h**) MRE % and its standard deviation in the three areas. (**i**) Linear regression between histology scores and (1) measured peak temperature values (PTM-blue curve), and (2) predicted peak temperature values (PTP-red curve) for the three classes. The parameters resulting from the correlation are also reported.

The TDSM was developed to automatically categorize pixels in the ROI into “no damage” (yellow), “ring” (black), and “thermo” (red). Optical information provided by HSI was used to train TDSM together with annotated RGB images providing the ground truth damage (Figure 6a). RGB images with predicted and ground truth damage zones are shown in Figure 6b for the ten acquisition steps. The TDSM model starts performing better for acquisition steps above 70 °C, namely a temperature increase inducing detectable thermal damage. According to the images, the area of LA-induced damage (“ring” + “thermo”) expands at the start of the procedure and after 70 °C its perimeter is not subjected to consistent variation. As the LA procedure progresses, the “thermo” area starts appearing and it enlarges until 110 °C. On the other hand, the number of pixels belonging to the “ring” class decreases until the end of ablation. 

TDSM performance is reported as DICE and accuracy metrics in Figure 6c,d. Concerning the DICE metric (Figure 6c), the “thermo” class had a high score that exceeded 0.86 after 80 °C. Hence, only a few pixels have been annotated as a carbonized zone in the starting steps and they are not predicted until the maximum temperature of 80 °C occurs. Carbonization at 70 °C was visible in two tests and it was predicted by the TDSM only in one of them, thus leading to DICE of 0.27. At 60 °C, only one test had some pixels (2 out of 58) in the “thermo” zone which were not predicted by TDSM, giving DICE equal to 0.

For the “ring” class, values ranged from 0.81 at 110 °C to 0.92 at 70 °C, thus demonstrating high performances of the model when the laser-induced damage generates and progresses. Moving to the “no damage” class, the DICE score was 0.94 or higher, which indicates good predictions. Concerning classification accuracy, a mean value of 0.91 or above was obtained during the treatment, (Figure 6d). Given the lower number of pixels, the highest accuracy is found for the “thermo” class with a mean of 0.93 or above. Conversely, “ring” and “no damage” show very close results with a maximum of around 0.97 in the earlier stages of the procedure. 

Afterward, we trained the PTPM to predict the peak temperature map from a HS image using the measured peak temperature maps from the IR camera data (Figure 6e). Predicted peak temperature maps are shown with ground truth in Figure 6f in all the acquisition steps. Qualitatively, predictions are better for acquisition step above 80 °C and in the damaged areas. To evaluate performance quantitatively, two-dimensional Rel_*err*_ maps were generated for the “no damage”, “ring”, and “thermo” classes in Figure 6g. A maximum Rel_*err*_ of 45% was mainly present at 36 °C leading to the MRE value of around 20% (Figure 6h). At that temperature, indeed, no tissue damage occurs, and the optical response is not subject to relevant variation enabling pixel prediction. However, the highest MRE is measured in the “thermo” zone at 60 °C with an MRE of approximately 23%. Again, this is due to the lack of structural damage in the tissue for those starting temperature-time settings. Additionally, only a few pixels in one test belong to this class, therefore high error localized in this test affects more the total mean value. An MRE of around 19% is reported at 70 °C; again, this result is due to a limited number of tests showing the “thermo” class and the low performances in temperature prediction for such conditions. Once the irreversible tissue damage occurs for almost all the pixels in the ROI, the PTPM presents much better performance. After the 80 °C steps, the MRE was approximately 10% for all three classes. For the “no damage” and “ring” classes in the Rel_*err*_ maps, areas of higher error are mainly localized at the internal and/or external borders. This could be due to errors in the alignment process as well as to residual pixels holding specularities. In the final step, to better validate the PTPM, a linear correlation was applied between the histology scores and the measured and predicted peak temperature values in the three classes (Figure 6i). Results show r_measured_ and r_predicted_ of 0.90, *p* < 0.0001 and r2 = 0.82 in both cases. No statistical difference was found between the curves (*p* = 0.7495), confirming the PTPM predictions as accurate.

**Figure 7:**
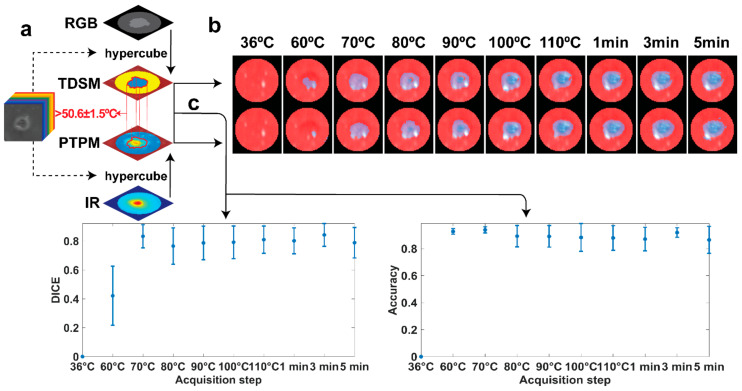
(**a**) Scheme demonstrating the approach used to compare the models to define the HSI abilities in margins detection. (**b**) Masks of damage for the two models overlayed on the RGB images in the ROI for all the acquisition steps of the first test. (**c**) Mean values and standard deviation for DICE and accuracy parameters defining the match between the two model.

As a final step, the ability of the HSI tool to detect the ablation margin was measured by combining the results of the two models, Figure 7a. The margins found with the TDSM were compared with the ones extracted from the PTPM. In the first case, margins were automatically defined from the TDSM classifying pixels in the ROI as total damage including “thermo” and “ring” classes. In the second case, margins were obtained by segmenting the predicted peak temperature maps using a threshold of 50.6 ± 1.5 °C. The selection of the threshold is explained in the Appendix A. Masks of damage obtained in both cases are overlayed on the RGB images in the ROI, Figure 7b. Quantitative comparison of thermal damage predictions by the TDSM and PTPM using DICE and accuracy scores are shown in Figure 7c. DICE scores are approximately 0.80 after the acquisition at 70 °C, with a minimum value of 0.76 at 80 °C. The low ability in detecting correct temperature values when tissue is not irreversibly damaged affects the PTPM mask of damage at 60 °C. Thus, a very low DICE of 0.42 is found at 60 °C. On the other hand, accuracy exceeds 0.87 for all the acquisition times, indicating good agreement.

## 4. Discussion

The need for intraoperatively LA monitoring has been largely debated [12,40]. Currently, the lack of real-time control of the therapy outcome is one of the major limitations for its application as a clinical standard. In this study, we propose a new HSI-based paradigm using CNN to monitor and predict the LA procedure effect. Starting from the damage assessment provided by the histology as previously described [41], two CNNs were trained with supervised learning using a combination of HS and thermal data in an experimental model of in vivo porcine liver. The CNN-based predictions showed promising performances for acquisition steps >80 °C in the three thermal areas: “thermo”, “ring”, and “no-damage”. High DICE and accuracy, and MRE with a maximum value of ~11% were found for TDSM and PTPM, respectively. On the other hand, by excluding the “thermo” class, high values for DICE and accuracy are measured for all the acquisition steps with an MRE ≤ 12%. A proper time-temperature combination, indeed, is needed to successfully discriminate the “thermo” class with HS tool. Additionally, a high correlation between histology scores and measured and predicted peak temperatures suggested that HSI can depict the laser-induced damage. No statistical difference was found between the two linear regression lines, confirming the robustness of the PTPM. Considering the importance of defining the ablation margin in clinical practice [42], we tested the ability of HS tool in depicting the damage margins using the area of damage predicted from the two models. The comparison between the two models’ outcomes gave high DICE and accuracy values for acquisition step >60 °C. The good agreement demonstrates the capacity of delineating the ablative margins for PTPM. Therefore, our study demonstrates the feasibility of peak temperature (50.6 ± 1.5 ºC) CNN-based prediction for the non-reversible damage detection. It is worth noting that the extracted temperature threshold of ~50 °C has been recognized as the threshold of coagulative necrosis [2].

In the present study, peak temperature (the maximum temperature experienced by the tissue during the procedure) was set as a model for thermal damage outcome. Therefore, we defined the peak temperature map as quantitative and reliable information demonstrating good performances after 60 °C (see the Appendix A). Additionally, the high correlation with histology further validated its use as a thermal damage model thus overcoming the limitation of common thermal models (e.g., CEM_43_, Arrhenius) which use empirical constants and rely on simplifying hypotheses of the complex thermal damage phenomena [15,18]. Besides HSI potentiality, an important limitation in its usage as a monitoring tool is the relatively long acquisition time. Rapid scanning and processing are necessary for dynamic targets or real-time feedback of therapy outcomes. Additionally, in vivo monitoring of internal body cavities requires a tool that can easily reach the target in a minimally invasive manner. In our experimental model, the treatment is made in “open surgery” due to the lack of an integrated endoscopic tool. Nevertheless, snapshot HS video-endoscopes and percutaneous hyper/multispectral catheters are in the development stage for future clinical use [43,44]. Another drawback is the presence of artifacts due to specular light, mainly due to the bright and wet liver surface, and the need for proper scene illumination. Concerning the first point, the pixels of specularity were excluded from the analysis. To further reduce specular reflections, cross-polarization scheme could be used in the future [45]. However, these aspects could be minimized by an endoscopic approach. The superficial approach allowed visual control and IR camera use to monitor the procedure. IR thermography has been demonstrated for diagnostic and real-time monitoring in medicine [46,47]. Additionally, the robustness and high accuracy justify its use for providing the supervision information to train the PTPM. It is also worth noticing that we chose to use the laser wavelength of 808 nm, to allow high penetration of the laser beam in the tissue [48]. Considering the HS camera working range, to avoid misleading peaks in the data, the laser current was controlled under the acquisition step and switched off during the HS acquisition time. Thus, the thermal damage induced was then an effect of non-continuous treatment. Specific lens filtering of the wavelength of interest could be used in the future to address such limitations. 

Overall, in our study, PTPM demonstrated an optimal agreement with the TDSM in the margin detection providing a consistent threshold for the margin with high accuracy [2]. At the same time, the high correlation with the histology score of peak temperature predicted and measured, confirmed that PTPM preserves the temperature information given by the IR camera accordingly with the histopathological assessment. This feature represents an important finding and a step forward over the current techniques, such as the MRTI, in which direct correlation with actual thermal damage induced is still under investigation. Additionally, PTPM, contrary to the TDSM, was trained using thermal data without the need for a human annotator, thus saving time and demonstrating the future chance of moving towards a completely automated HSI-based approach. 

## 5. Conclusions

This preliminary investigation furnishes robust evidence that PTPM is a suitable paradigm for the prediction of liver LA damage intraoperatively using a unique camera and analysis, potentially overcoming many barriers to the current thermal damage monitoring in LA and other thermal therapies. In the future, different CNN architectures should be compared on this problem and their benefit over ‘classical’ ML models, such as Support Vector Machine and Support Vector Regression, should be studied. Moreover, the potential of the proposed prediction model in also discriminating the “thermo” margin and different types of damage could be investigated. In this context, PTPM could be used to predict the future area of damage after the regenerative process in a survival study. Future works should involve the use of tumor tissue to evaluate the consistency of the proposed approach for a clinical scenario. Nevertheless, as a first in vivo validation, the use of normal liver tissue was considered acceptable as a proof of concept of the whole paradigm. Lastly, the ability of HS to detect tumor lesions may be in the future combined with the ablation area detection, for a theranostic and selective treatment localized to the cancer cells.

## Figures and Tables

**Table 1:** CNN architectures.

Layer	Kernel Shape		Number of Output Channels		Stride		Number of Trainable Parameters	
Conv1	(3,3,3)		20		(1,1,1)		560	
ReLU	/		/		/		/	
Pool1	(3,1,1)		20		(2,1,1)		1220	
Conv2	(3,3,3)		35		(1,1,1)		18,935	
ReLU	/		/		/		/	
Pool2	(3,1,1)		35		(2,1,1)		3710	
Conv3	(3,1,1)		35		(1,1,1)		3710	
ReLU	/		/		/		/	
Pool3	(2,1,1)		35		(2,1,1)		2485	
ReLU	/		/		/		/	
FC	TDSM	PTPM	TDSM	PTPM	TDSM	PTPM	TDSM	PTPM
	(455,3)	(455,1)	3	1	/	/	1368	456

**Figure A1:**
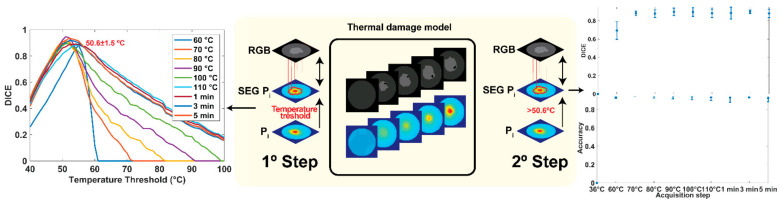
Schematic of the analysis performed and metrics calculated to validate peak temperature maps as a thermal damage mode.

## Data Availability

The data presented in the study are available on request from the corresponding author.

## Appendix A

To demonstrate the validity of the peak temperature maps (P_*i*_) extracted from the IR video to be used as a thermal damage model, the following two steps were carried out (see Figure A1).

Step 1: RGB images manually annotated were used to find the temperature threshold detecting the damage in the P_*i*_. The optimal threshold to segment the P_*i*_ was selected considering the maximum DICE. Then, a single global threshold was obtained averaging among the results obtained in each test. This value was found to be 50.6 ± 1.5 °C.

Step 2: The threshold was used to segment the Pi to finally detect ablation margins. DICE and accuracy parameters were then calculated by comparing the obtained damage with the ground truth (RGB images manually annotated) and showed values higher than 0.93 and 0.87 (for temperature >70 °C) for accuracy and DICE, respectively. At 60 °C, a lower DICE of 0.69 was measured because for temperatures ≤60 °C the thermal damage is not instantaneous. Therefore, P_*i*_ can be considered a valid thermal damage model, holding temperature and time info, especially after reaching 60 °C.
